# High Reproducibility of Histological Characterization by Whole Virtual Slide Quantification; An Example Using Carotid Plaque Specimens

**DOI:** 10.1371/journal.pone.0115907

**Published:** 2014-12-26

**Authors:** Joyce E. P. Vrijenhoek, Bastiaan G. L. Nelissen, Evelyn Velema, Kristy Vons, Jean-Paul P. M. de Vries, Marinus J. C. Eijkemans, Hester M. den. Ruijter, Gert Jan de Borst, Frans L. Moll, Gerard Pasterkamp

**Affiliations:** 1 Experimental Cardiology Laboratory, University Medical Center Utrecht, Utrecht, the Netherlands; 2 Interuniversity Cardiology Institute of the Netherlands, Utrecht, the Netherlands; 3 Department of Vascular Surgery, University Medical Center Utrecht, Utrecht, the Netherlands; 4 Department of Vascular Surgery, St. Antonius Hospital Nieuwegein, Nieuwegein, the Netherlands; 5 Julius Center of Health Sciences and Primary Care, University Medical Center Utrecht, Utrecht, the Netherlands; Baker IDI Heart and Diabetes Institute, Australia

## Abstract

**Objective:**

Tissue biobanks are an important source for discovery and validation studies aiming for new proteins that are causally related with disease development. There is an increasing demand for accurate and reproducible histological characterization, especially for subsequent analysis and interpretation of data in association studies. We assessed reproducibility of one semiquantative and two quantitative methods for histological tissue characterization. We introduce a new automated method for whole digital slide quantification. Carotid atherosclerotic plaques were used to test reproducibility.

**Methods:**

50 atherosclerotic plaques that were obtained during carotid endarterectomy were analysed. For the semiquantitative analysis, 6 different plaque characteristics were scored in categories by two independent observers, and Cohen's κ was used to test intra- and interobserver reproducibility. The computer-aided method (assessed by two independent observers) and automated method were tested on CD68 (for macrophages) and α smooth muscle actin (for smooth muscle cells) stainings. Agreement for these two methods (done on a continuous scale) was assessed by intraclass correlation coefficients (ICCs).

**Results:**

For the semiquantitative analysis, κ values ranged from 0.55 to 0.69 for interobserver variability, and were slightly higher for intraobserver reproducibility in both observers. The computer-aided method yielded intra- and interobserver ICCs between 0.6 and 0.9. The new automated method performed most optimal regarding reproducibility, with ICCs ranging from 0.92 to 0.97.

**Conclusions:**

The analysis of performance of three methods for histological slide characterization on carotid atherosclerotic plaques showed high precision and agreement in repeated measurements for the automated method for whole digital slide quantification. We suggest that this method can fulfill the need for reproducible histological quantification.

## Introduction

Biobanking of human tissues is an important cornerstone in the discovery and validation of causally related determinants of life-threatening diseases. Genotyping studies of human DNA are characterized by stringent quality controls. However, accurate phenotyping of a diseased patient and biosamples is becoming a major hurdle for the assessment of geno-phenotypic associations. Dissected tissues collected in biobanks are a great asset for phenotyping diseases and for prognostic studies, in which histological characterization is widely applied. It is evident that accuracy and reproducibility of histological characterization is key for optimal phenotyping of human tissues and subsequent interpretation of data for association studies.

An example of a research field where phenotyping by histological characterization is commonly executed is the characterization of atherosclerotic plaques, where stable and unstable lesions are differentiated based on inflammatory, lipid, and fibrous components. Previously, assessment of semiquantitative scoring (SQ) of different carotid plaque characteristics indicated moderate to good reproducibility as indicated by Cohen's kappa (κ) values [1], [2]. Still, further improvement regarding intra- and interobserver variability is required. Also, in ongoing longitudinal studies, a continuing check of reproducibility is essential. Furthermore, though variability of semiquantitative scoring based on subjective scoring within a laboratory could be acceptable, it may be difficult to extrapolate this other external studies. In atherosclerotic plaques, a computer-aided method (further annotated as method Q1) to score inflammatory cells and smooth muscle cells quantitatively was previously implemented to improve reproducibility that indeed performed well [2]. However, this method requires the user to manually set color thresholds for the positively stained areas within subjectively selected regions of interest.

Therefore, a targeted method to quantify characteristics in total tissue specimen is needed. Whole slide virtual imaging can be used for this purpose [3], [4]. To quantify characteristics on these whole slide images, we have developed the slideToolkit software (further annotated as method Q2). This is a new method using free open-source software, targeted to overcome the aforementioned limitations. We tested the precision (not the accuracy) of this automated method in the Athero-Express biobank, comprising carotid artery plaque specimens. The aim of this study was 1) to reassess intra- and interobserver variability of carotid plaque scoring within our biobank with the methods used before and 2) to evaluate the performance and precision of the new method Q2.

## Methods

### Sample selection

50 carotid plaque specimens were randomly selected from carotid endarterectomy (CEA) patients who were included in the Athero-Express study between 2002 and 2012 [5]. 68% of these patients was male with a mean age of 69 years. 78% was symptomatic with median time from event to CEA of 38 days. All patient characteristics are shown in table 1.

**Table 1:** Patient characteristics.

Male sex	68%
Age (mean) (SD)	69 (10)
Current smoking	39%
Diabetic mellitus	22%
Hypertension	72%
Body Mass Index (mean) (SD)	26 (3.6)
Hypercholesterolemia	54%
History of coronary artery disease	32%
History of peripheral intervention	24%
Preoperative acetylsalicyl acid use	96%
Preoperative statin use	69%
Total cholesterol (mean) (SD)	4.7 (1.3)
HDL (mean) (SD)	1.1 (0.35)
Clinical presentation	
* Asymptomatic*	22%
* Ocular symptoms*	27%
* Transient ischemic attack*	33%
* Stroke*	18%
Event to operation time (median) (IQR)	37.5 (18.5–70.3)
HDL: high density lipoprotein. IQR: interquartile range.	

### Ethics Statement

The Athero-Express study was approved by the institutional review boards of both participating hospitals (University Medical Center Utrecht, Utrecht, The Netherlands, and St. Antonius Hospital, Nieuwegein, The Netherlands) and patients gave written informed consent.

### Plaque processing

Plaques were removed during CEA and immediately processed in the laboratory, were the culprit lesion with a length of 5 mm was fixed in 4% formaldehyde, subsequently followed by decalcification and embedding in paraffin. 5 µm cross-sections were sliced and routinely stained for different characteristics: lipid core size (hematoxylin and eosin (HE) and Picrosirius Red with polarized light when appropriate), macrophages (CD68, Clone KP1, Novacastra reagents, Leica Biosystems, Rijswijk, the Netherlands), smooth muscle cells (α smooth muscle actin antibody, Clone 1A4, Sigma-Aldrich, Zwijndrecht, the Netherlands), collagen (picrosirius red), calcification (assessed using HE), and thrombus (HE and fibrin) [5]. CD68 and SMA were visualized with DAB (3,3′-diaminobenzidine), and were used in quantitative analyses in this study (methods Q1 and Q2). Other characteristics were not included in the quantitative analyses in this study.

### Semiquantitative analysis (method SQ)

Plaques were categorised in no, minor, moderate and heavy staining for most characteristics, except for lipid core (no, <40%, >40%) and overall phenotype (fibrous, fibroatheromatous and atheromatous). Scorings were done by two observers (EV and GP), both at 2 timepoints with at least a one-month interval. They were blinded for each other's scoring, their previous scoring (at the second observation), and for patient characteristics.

### Quantitative analysis, computer-aided with visual interpretation (method Q1)

In the CD68 three representative regions of interest of the plaque (excluding lumen and media) were selected at ×40 magnification according to decision of the same experienced technician over time (observer EV). The area occupied by DAB staining was determined by manually selecting colour thresholds by visual interpretation, for each of the three regions of interest separately. Thereafter, the total field occupied by tissue was calculated as a percentage of total area occupied by plaque on each region of interest (analySIS FIVE, Olympos soft imaging solutions). The mean percentage of three fields was taken as the final value. Scorings were done by observer 1 (KV) and observer 2 (TB), both at two timepoints with a one-month interval. They were blinded similarly as noted above, and also for the results of semiquantitative scorings.

### Automated quantitative analysis (method Q2)

**Figure 1:**
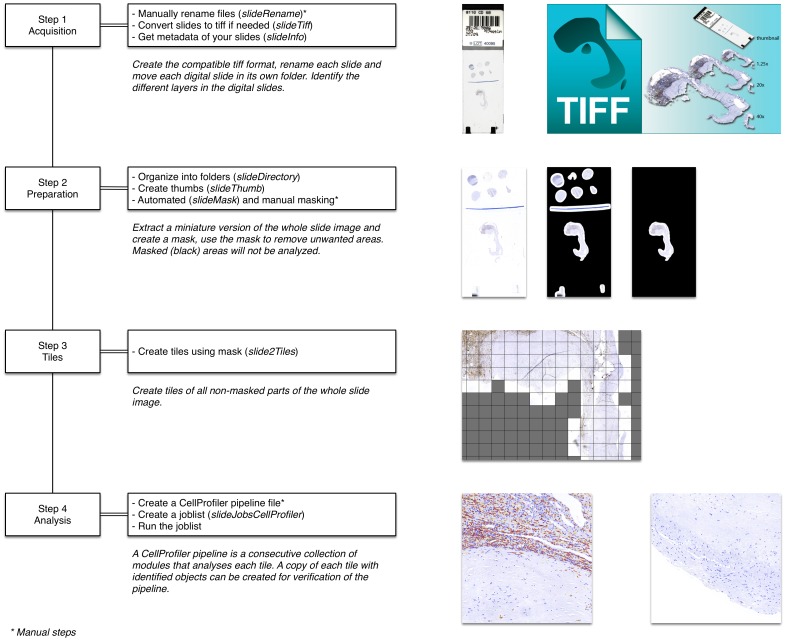
A common slideToolkit workflow consists of ‘Acquisition’, ‘Preparation’, ‘Tiles’ and ‘Analysis’.

For this analysis, histological slides were scanned in total using a Roche VENTANA iScan HT slide scanner. Each virtual slide was stored as a multi-page pyramid tiff using 90% JPEG compression. In short, a Q2 workflow consists of four consecutive steps. In the first step (acquisition), whole slide images are collected and converted to TIFF files. In the second step (preparation), files are organized. The third step (tiles), creates multiple manageable tiles to count. In the fourth step (analysis), tissue is analyzed and results are stored in a data set (Fig. 1 in S1 Methods and pipelines in S1 and S2 Data for details). As a part of this method, actual image analysis was done using CellProfiler (version 2.1.0.Beta_2a.linux, Cellprofiler Cell Image Analysis Software, Broad Institute, Cambridge, Massachusetts, USA). CellProfiler uses predefined pipelines to analyze and measure histological images. A pipeline is a sequential series of modules that each performs an image processing function. For example, to measure the DAB positive cells, the following was done during the analysis step. First we used a module to convert the image into two separate gray scale images. One gray scale image was analyzed for HE, and only HE was white (positive), the rest was black (negative). We did the same for DAB, where DAB was white (positive) and the rest was negative (black). To find all nuclei within DAB positive areas, we used a module to use the DAB gray scale image as a mask for the HE gray scaled image. In this way it is possible to only analyze the HE positive nuclei within DAB positive areas. Finally, to identify these nuclei within DAB positive areas, a module to identify objects was used to find HE nuclei sized between 8 and 40 pixels. Hence, method Q2 differs from Q1 in that Q2 analysis the whole slide, so it has no selected regions of interest, and thresholds and measurements settings are set before analysis. More details on how method Q2 works and the accompanying pipelines can be found in S1 Methods and S1 and S2 Data.

For CD68 and SMA stained slides, separate CellProfiler pipelines were created. For macrophages, two parameters were calculated: total DAB positive nuclei as a ratio of total plaque area (macrophage nuclei), and total DAB positive area as a ratio of total plaque area (macrophage area). For smooth muscle cells, only the area was calculated (smooth muscle cell area).

### Statistical analysis

Reproducibility of different scoring methods was assessed by different measures of agreement, as shown in table 2. Intra- and interobserver variability was assessed within the same methods, except for method Q2 that only includes intraobserver variability.

**Table 2:** Statistical tests used for analyzing agreement of different plaque scoring methods.

	SQ	Q1	Q2
**SQ**	Cohen's κ	NC	NC
**Q1**	NC	ICC + Bland-Altman	NC
**Q2**	NC	NC	ICC + Bland-Altman

SQ: semiquantitative analysis; method Q1: quantitative analysis, computer-aided; method Q2: automated quantitative analysis; NA: not applicable; NC: not comparable (measurements are on different scales).

Variability of scorings done on a categorical scale (method SQ) was analyzed by Cohen's κ, with linear and quadratic weightings [6]. Values between 0 and 0.2 are generally indicated as slight agreement, 0.21–0.4 as fair, 0.41–0.6 as moderate, 0.61–0.8 as substantial, and 0.81–1 as almost perfect agreement.

Variability of scorings done on a continuous scale (method Q1 and Q2) was analyzed by calculating intraclass correlation coefficients (ICCs), by a two-way mixed, single measures model. Interobserver ICCs of method Q1 were calculated by using only the first measurements of both observers (otherwise we would calculate a composite of intra- and interobserver variability). To check if this approach was valid, a random effects model was fit using the “lme4” package in R statistics [7] with slide number, rater, and repetition as random effects. A fixed effect was not included, because there was only 1 method to be assessed. For both macrophages and smooth muscle cells, variance induced by repetition was less than 1% of the total variance.

For method Q2, there was only 1 observer (the computer). Therefore, only intraobserver ICC and no interobserver variability, was calculated. We did not calculate ICCs between measurements of different methods, because these were measured on different scales and had different ranges. Therefore, it was not possible to accurately define cutoffs or ranks within different ranges of measured values to compare the different methods. The different scales (categorical for SQ and continuous for Q1 and Q2) were also the reason why we could not use the same statistical methods for all scoring methods. For comparison between κ and ICC, quadratic weighted κ can be used, as this is expected to be equal to the ICC; quadratic weighted κ can be written as a ratio of the difference between expected and observed disagreement, divided by the expected disagreement. Since for quadratic weighted κ all disagreement terms contain quadratic differences between categorical numbers, after some mathematical manipulations as shown by Fleiss and Cohen, this is the same as the ratio of variances that defines the intraclass correlation coefficient [8].

In addition to ICCs, Bland-Altman plots were made to visualize the amount of (dis)agreement between the continuous scorings [9] using the ggplot2 and gridExtra packages in R statistics [7]. Because the data were right-skewed and the spread of differences increased with increasing mean of the observations, natural logarithmic transformed values were used in these plots.

SPSS version 20.0 (IBM Corp, IBM SPSS Statistics for Windows, Armonk, NY) was used to calculate all ICCs.

## Results

### Semiquantitative analysis (SQ)

For all plaque characteristics under study, κ values were calculated with linear and quadratic weights, as shown in table 3. In this results section we only report the quadratic weighted κ. Quadratic weighted κ can also be compared to the ICC, as explained previously.

**Table 3:** Intra- and interobserver variability of semiquantitative scoring of plaque histology.

				Intraobserver κ EV (95% CI)		Intraobserver κ GP (95% CI)		Interobserver κ (95% CI)
	Categories	Agreement	Linear weighted	Quadratic weighted	Linear weighted	Quadratic weighted	Linear weighted	Quadratic weighted
Lipid core	3	58%	0.58 (0.37–0.79)	0.65 (0.32–0.98)	0.65 (0.48–0.83)	0.68 (0.44–0.93)	0.44 (0.25–0.63)	0.55 (0.28–0.81)
Macrophages	4	57%	0.65 (0.48–0.82)	0.77 (0.51–1)	0.68 (0.56–0.81)	0.84 (nc)	0.56 (0.41–0.71)	0.69 (0.47–0.90)
Smooth muscle cells	4	46%	0.65 (0.45–0.85)	0.72 (0.20–1)	0.57 (0.38–0.76)	0.63 (0.27–0.99)	0.40 (0.23–0.57)	0.59 (0.31–0.87)
Collagen	4	79%	0.75 (0.59–0.91)	0.80 (0.33–1)	0.67 (0.50–0.84)	0.75 (0.35–1)	0.72 (0.56–0.88)	0.79 (0.41–1)
Calcification	4	40%	0.75 (0.63–0.88)	0.84 (0.66–1)	0.67 (0.53–0.80)	0.81 (nc)	0.42 (0.28–0.56)	0.59 (0.41–0.77)
Thrombus	4	42%	0.71 (0.54–0.880	0.76 (0.36–1)	0.54 (0.39–0.69)	0.72 (0.65–0.80)	0.38 (0.23–0.54)	0.55 (0.33–0.77)

nc: not calculated due to a substantial proportion of zeros in the crosstable. CI: confidence interval. EV and GP indicate different observers.

Inter- and intraobserver variability was moderate to substantial for all characteristics; intraobserver κs were between 0.65 to 0.84 and 0.63 to 0.84 for observers EV and GP respectively, for interobserver comparisons κs ranged between 0.55 and 0.69.

### Quantitative analysis, computer-aided with visual interpretation (method Q1)

For macrophage staining, median value of percentage of stained area of all observations (n = 200) was 0.46% (interquartile range (IQR): 0.11–1.1%). Intraobserver ICC for observer 1 was 0.92 (95% confidence interval (CI): 0.86–0.95) and for observer 2 0.84 (95% CI: 0.73–0.91). For smooth muscle cells, median value of percentage of stained area of all observations was 1.3% (IQR: 0.54–2.6%). Intraobserver ICC was 0.76 (95% CI: 0.61–0.85) and 0.86 (95% CI: 0.80–0.88), respectively. Interobserver ICCs were lower, with 0.71 for macrophages and 0.62 for smooth muscle cells (table 4).

**Table 4:** Intra- and interobserver variability of quantitative methods scoring of plaque histology.

	Macrophages, nuclei	Macrophages, area or %*	Smooth muscle cells
Q1, interobserver ICC	NA	0.71 (0.53–0.82)	0.62 (0.42–0.77)
Q1, intraobserver ICC observer 1	NA	0.92 (0.86–0.95)	0.76 (0.61–0.85)
Q1, intraobserver ICC observer 2	NA	0.84 (0.73–0.91)	0.86 (0.80–0.88)

Method Q1: quantitative computer-aided technique of selected areas with visual interpretation of color threshold; method Q2: automated quantitative analysis: whole slide imaging and automated quantitative analysis software; NA: not applicable (macrophage nuclei were not assessed in method Q1).

*For method Q1: percentage stained area of total plaque; for method Q2: total percentage stained area per total area in hematoxylin staining.

**Figure 2:**
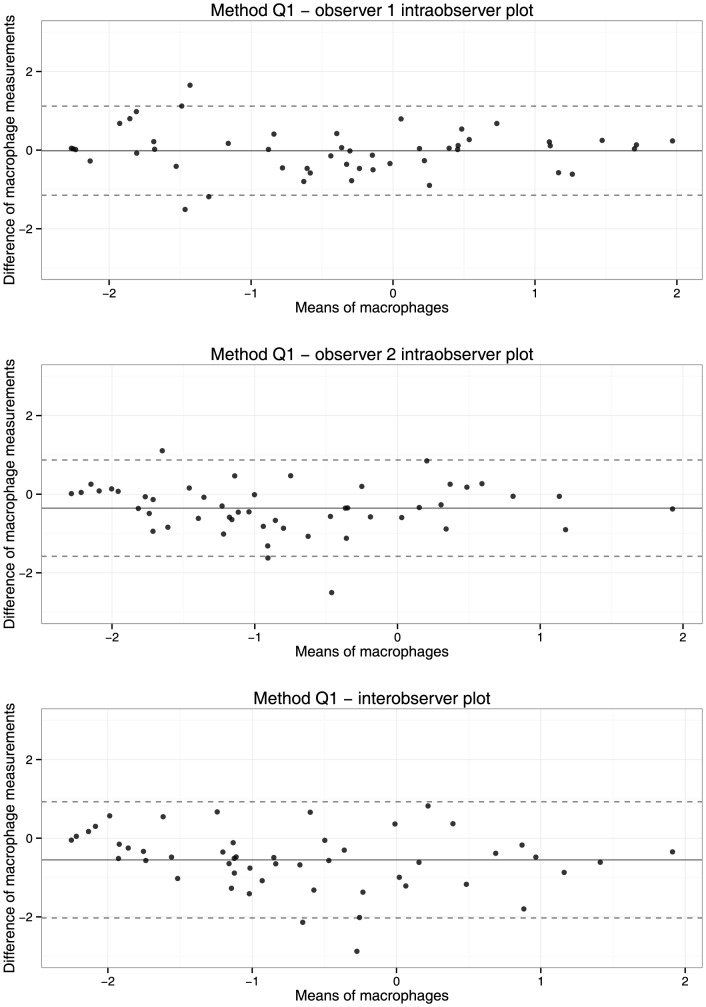
Bland-Altman plots of intra- (upper two panels) and interobserver (lower panel) reproducibility of method Q1 for measurement of macrophages. Measurements were logarithmically transformed after adding 0.1. The continuous line shows the mean difference of measurements, the dotted line indicates 95% limits of agreement.

**Figure 3:**
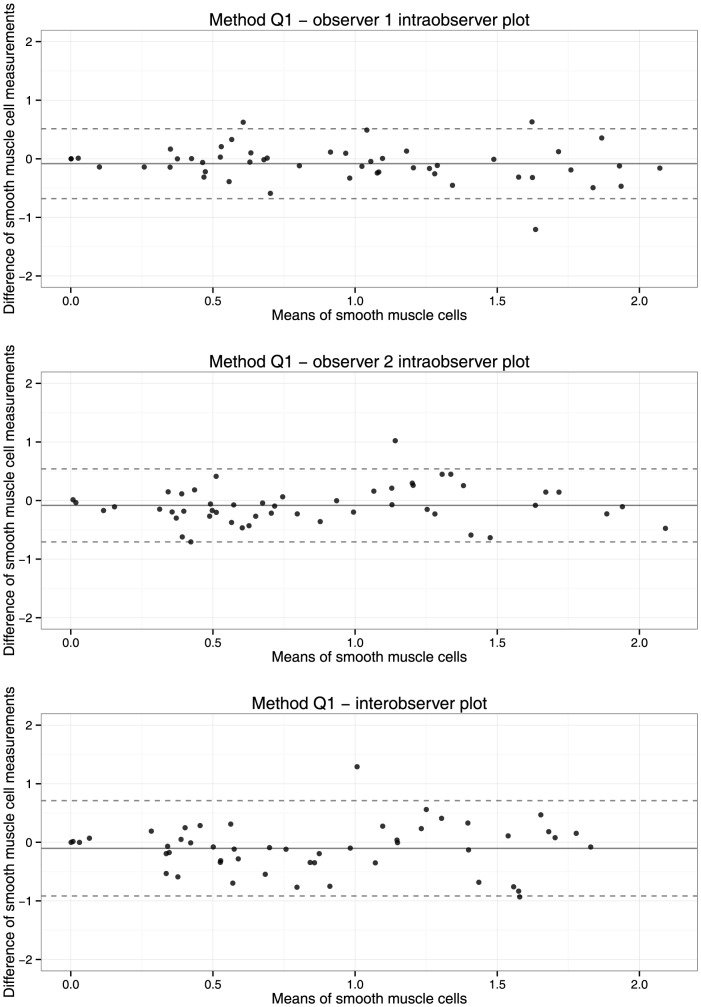
Bland-Altman plots of intra- (upper two panels) and interobserver (lower panel) reproducibility of method Q1 for measurement of smooth muscle cells. Measurements were logarithmically transformed after adding 1. The continuous line shows the mean difference of measurements, the dotted line indicates 95% limits of agreement.

Bland- Altman plots show that the variation in log transformed macrophage and smooth muscle cell measurements remains quite constant with increasing percentage staining, both for intra- and interobserver measurements (Figs. 2 and 3).

### Automated quantitative analysis (method Q2)

For the ratio of macrophage nuclei and area per total plaque area, median level of all observations (n = 91, remaining 9 scorings were missing) was 6.0 * 10^5^ (IQR: 2.3×10^5^–9.8×10^5^), and 0.065 (interquartile range (IQR): 0.030–0.14), respectively. Intraobserver ICC for macrophage nuclei was 0.97 (95% CI: 0.94–0.98) and for macrophage area 0.97 (95% CI: 0.95–0.98).

Median level of smooth muscle cell area of all observations (n = 95) was 0.32 (IQR 0.21–0.47). ICC of smooth muscle cell scoring was 0.92 (95% CI: 0.86–0.95) (table 4).

**Figure 4:**
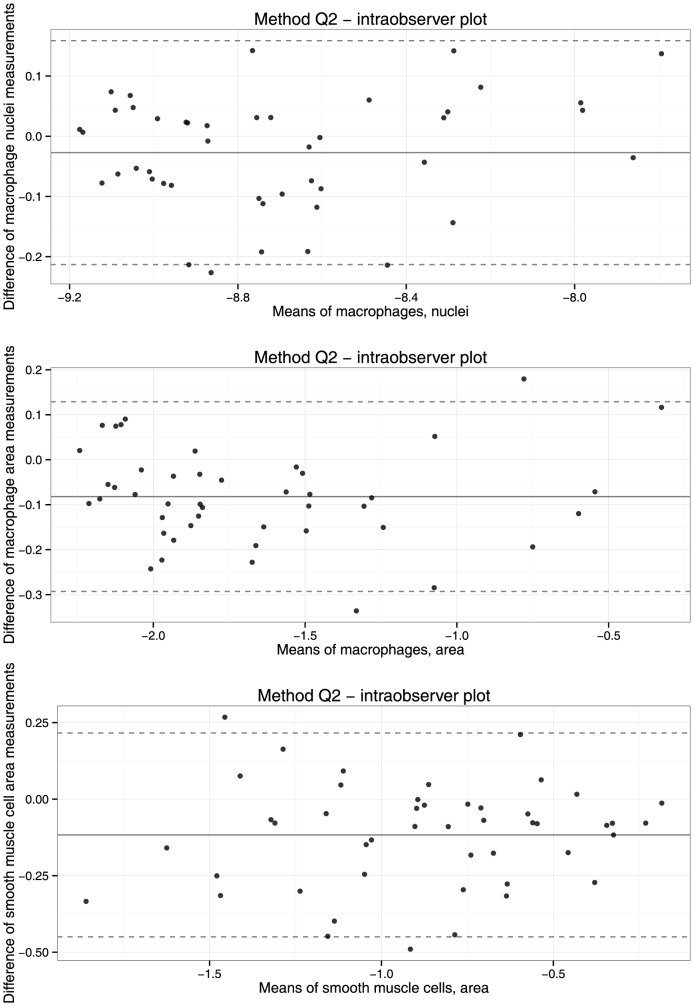
Bland-Altman plots of intraobserver reproducibility of method Q2 for measurement of macrophage nuclei (upper panel), macrophage area (middle panel), and smooth muscle cell area (lower panel). Measurements were logarithmically transformed after adding 0.0001, 0.1, and 0.1, respectively. The continuous line shows the mean difference of measurements, the dotted line indicates 95% limits of agreement.

Again, Bland-Altman plots suggest that the variation in log-transformed macrophage and smooth muscle cell measurements remains constant across the range of all measurements (Fig. 4).

## Discussion

Here we report that a method to quantify tissue histology fully automatically can be executed with high reproducibility. We used macrophage and smooth muscle cell infiltration in carotid atherosclerotic plaques as markers to test this method. If we compare this method to other (semi)quantitative techniques that have been applied previously, this new quantitative method seems to perform better regarding ICC values of two repeated measurements, with narrower 95% confidence interval ranges. The high precision suggests that quantification by this technique allows for more reliable association studies, compared to traditional methods using semiquantitative analysis that showed modest to substantial reproducibility in this study, as shown before [2]. This indicates that characteristics that require visual interpretation may suffer from variability among researchers and research centers, and should be interpreted with caution.

### Applications in research

There are different research applications in which reproducibility of atherosclerotic plaque histology is important. First, plaque imaging (by MRI) is increasingly used as a technology to detect vulnerable plaque characteristics noninvasively, for example for associations with symptoms and/or future cardiovascular events [10]–[13]. For validation of imaging techniques, comparison with atherosclerotic plaque histology is necessary. Measured variability between plaque histology and plaque imaging for example, is a result of the added variability of both methods. Second, human genotyping studies are emerging and applied to study causality of disease and for discovery of potential therapeutic targets. Therefore, accurate and reproducible association of phenotypic tissue characteristics with genetic data is relevant, which requires improved phenotyping of tissues and disease. This objective applies for all diseases where histological phenotyping is an issue and goes beyond the scope of atherosclerotic disease. Phenotypic differences in disease presentation between subjects (such as differences between macrophage infiltration in atherosclerotic plaques) are a result of real variation, and ‘noise’: variability within and between observers using a certain technique. Studies on genetic variability are characterized by high qualitative output and reproducibility and the large variability in phenotypic outcomes is a major challenge before we can optimally assess and interpret genotypic-phenotypic associations.

### Applications in clinical practice

Another application, in which similar digital imaging techniques are already in use in some clinical laboratories, is assessing Her2 receptor status in breast cancer by automated cellular imaging system III (ACIS III, Dako). Standardized scoring protocols using this digital image analysis system have been tested and showed better concordance with fluorescence in situ hybridization, compared to manual semiquantitative interpretation of immunohistochemical slides [14]. Drawbacks of the ACIS III system is that the algorithm for the color threshold is fixed, so that changes in staining quality will influence scoring, and that this software only works on specific staining kits from the same company (like HercepTest).

### Strengths and limitations

This study comprised a random sample of carotid plaques, and a comprehensive analysis of two prior techniques and a new method to indicate performance of these different techniques. The new method (method Q2) uses whole slide imaging and thus contains all the information that is available in the tissue, in contrast to only a selection of tissue (method Q1) that may not reflect the total plaque. Other advantages of method Q2 are that it can be used on regular slides, and is fast and efficient due to parallel processing, which makes it possible to handle large numbers of slides simultaneously.

While we show high precision of method Q2, accuracy could not be assessed, as there is no gold standard to quantify plaque histology. Measuring accuracy is a challenge, because it remains unknown what the ‘real’ quantity of a certain marker is. We can only try to approach this ‘true’ quantity as good as possible by assessing reproducibility of repeated measurements, which is a measure of precision. Furthermore, precision is also dependent on which marker is used to visualize cells in tissue specimens, and measurements may accordingly be different. We tested only CD68 as a marker for macrophages, and α smooth muscle actin as a marker for smooth muscle cells.

There are several reasons why reproducibility remains an issue in an automated system such as Q2. First, we found that some slides contained undesired artefacts, like airbubbles, pen markings or positive antibody control tissue. When analyzing slides semiquantitatively or when manually selecting areas of interest (Q1), these artefacts are non-concerning. However, during whole slide analysis (Q2), these artefacts can result in false measurements. We created masks to exclude these parts of the whole slide images. Masks can be created automatically, but often these masks need to be altered by a human to remove unwanted artifacts (pen markings etc.) and this can theoretically influence reproducibility. In addition, different circumstances could occur while creating the digital image bij the scanner (dust, lack of focus, increased lighting, etc.), which could also result in different measurements.

Q1 and Q2 were not applied for quantifying other plaque characteristics. This is because Q1 was only found to be feasible on DAB-stained sections, and the application of Q2 has started on stainings visualized by DAB, with pipelines for the other characteristics still in development. Other stainings and/or antibodies may also show different results, and our antibodies may not always be the most optimal. However, as discussed before, this study did not aim to study accuracy of the antibodies, but focuses on reproducibility and application of a new scoring method on currently used stainings. This means that we do not yet present a standardized technique, however in the future consensus may be reached on what the most optimal method for tissue quantification should be, and good precision of this quantification method is a good starting point. In addition, we cannot yet generalize the applicability of our new quantitative method to other laboratories, and tissue types. Therefore, with every new application, reproducibility of scoring methods used on specific tissue specimens should be tested first. Pipelines for other stains are currently under development. Finally, we only analyzed reproducibility of the culprit lesion and cannot make inferences regarding reproducibility of other plaque segments. However, previous research by our group has shown that spatial differences in the plaque are minimal [2].

In the future, histological tissue quantification has to remain subject to ongoing reproducibility assessment and necessary updating, as computer-aided technology is in continuous development, and variability in tissue examination may change over time. The use of open source software makes frequent releases and updates possible [15]. It is to be expected that digitizing slides will become increasingly common, making automated quantification such as method Q2 cheaper and more accessible.

In conclusion, the (semi)quantitative methods currently studied to analyze plaque histology perform well, according to intra- and interobserver variability. The new automated method (Q2) presented here, using whole slide imaging and open source software, showed high precision and agreement in repeated measurements. This suggests that this technique can be used to reliably quantify tissue histology for research purposes.

## Supporting Information

S1 Methods **Methods of the slideToolkit.**
(DOCX)

S1 Data **CellProfiler pipeline (.cp file) for CD68 staining for quantification of macrophage area and nuclei.**
(CP) Click here for additional data file.

S2 Data **CellProfiler pipeline (.cp file) for smooth muscle cell actin staining for quantification of smooth muscle cell area.**
(CP) Click here for additional data file.
