# Supplementary material for: High Reproducibility of Histological Characterization by Whole Virtual Slide Quantification; An Example Using Carotid Plaque Specimens
Source: PLoS One. 2014 Dec 26;9(12):e115907. doi: 10.1371/journal.pone.0115907 (PMC4277457; doi:10.1371/journal.pone.0115907)
Supplement: S1 Methods — Methods of the slideToolkit. (DOCX) [file pone.0115907.s001.docx]

**SUPPORTING METHODS S1.**

**The slideToolkit method**

The slideToolkit is a collection of open source libraries and scripts to handle each step from virtual slides to the storage of your results. The toolkit is developed for modern (2014) personal computers (running *nix system[Linux, OS X, Unix]) and high-performance computing (HPC) systems. A common slideToolkit workflow consists of four consecutive steps. In the first step, “acquisition”, virtual slides are collected converted to .TIFF files. In the second step, “preparation”, files are organized for future steps. The third step, “tiles”, creates multiple manageable tiles to count. The fourth step, “analysis”, is the actual tissue analysis and saves the results in a meaningful dataset. These steps are schematically depicted in figure 1 of the main article.

**Step 1 - Acquisition**

Most slide scanners are, in addition to their own proprietary format, capable of storing the virtual slides in pyramid TIFF files. The slideToolkit uses the Bio-Formats library to convert other microscopy formats into the compatible pyramid TIFF format if needed. TIFF is a tag-based file format for raster images. A TIFF file can hold multiple images in a single file, this is known as a multi-layered TIFF. The term "Pyramid TIFF" is used to describe a multi-layered TIFF file that wraps a sequence of raster images that each represents the same image at increasing resolutions (figure 1). The different layers contain, among others, the slide label and multiple enlargements of the tissue on the slide.

To read whole slide images we use the opensource libTIFF libraries and the OpenSlide libraries. These libraries are also used to extract metadata (e.g. scan time, magnification and image compression) of the scanned slides. For image processing we use ImageMagick (ImageMagick 6.8.7-0 2013-10-16 Q16 http://www.imagemagick.org). ImageMagick is a command line image manipulation tool that is fast, highly adjustable and capable to handle big pyramid TIFF files. Descriptive information about the slide is stored as metadata and contains, for example, pixels per micrometer, presence of different layers, and scan date.

**Step 2 - Preparation**

In the following steps we create multiple output files for each slide. For each virtual slide a staging directory is created in which the virtual slide and all output data concerning the slide will be stored.

In digital image manipulation, a mask defines what part of the image will be analyzed and what part will be hidden. Usually a mask can be defined as black (hidden) or white (not hidden). We will use this technique to mask unwanted areas. The slideToolkit automatically creates a mask using a miniature version of the virtual slide (in our example this is layer 6 of the multi layered TIFF). Generated masks can be adjusted manually in an image editor of choice (such as the freely available GNU Image Manipulation Program; GIMP (http://www.gimp.org)), sometimes this is necessary to remove unwanted areas on the virtual slide (like marker stripes or air bubbles under the coverslip). Only non tissue parts of the virtual slides were masked.

**Step 3 - Tiles**

Working with whole 20x representations of the digitized slides is currently not possible due to hardware limitations. The goal of this step is to create multiple smaller images (i.e. tiles) from a whole slide. An upscaled version of the mask is placed over the 20x image of the slide (in our example this is layer 3 of the multi layered TIFF). Image manipulation on 20x sized virtual slides requires large amounts of computer RAM. To make it possible for computers without sufficient RAM to handle these files, the slideToolkit uses a memory-mapped disk file of the program memory. Using generated disk mapped memory files, the slideToolkit can efficiently extract all tiles. When no mask is used the slideToolkit uses a faster and more efficient way to create tiles without the use of a memory-mapped disk file.

**Step 4 - Analysis**

At this step, multiple tiles containing tissue data have been made, and the different objects in this tissue will be identified. CellProfiler is designed to quantitatively measure phenotypes from thousands of images automatically without training in computer vision or programming. CellProfiler can run using a graphical user interface (GUI) or a command line interface (CLI). Using the CellProfiler’s GUI, different algorithms for image analysis are available as individual modules that can be modified and placed in sequential order to form a pipeline. Such a pipeline can be used to identify and measure biological objects and features in images. Pipelines can be stored and reused in future projects. We created two pipelines for CD68 and SMA using the CellProfiler GUI and used CLI to run these. Both pipelines can be downloaded as .cp files, added as Supplementary data (“CD68_pipeline.cp” and “SMA_pipeline.cp”). An illustrated example on how to create pipelines in CellProfiler is described by Vokes and Carpenter.[1]

*CellProfiler CD68 pipeline*

This pipeline analyses the surface area and amount of CD68-DAB-positive cells compared to hematoxylin (HE) surface area. Each image was processed using the ‘UnmixColors’ module allowing the DAB stain and HE stain to be extracted into two channels. In the DAB channel, surface area was defined using the 'ApplyThreshold' module and included the ‘Otsu Global’ and the ‘three classes’ options. Weighted variance was minimized and pixels in the middle intensity class assigned to the foreground. The threshold correction factor was entered as ‘1.3’ and the lower and upper bounds on the threshold were ‘0.1’ and ‘1.0’ respectively. In the HE channel, surface area was defined using the 'ApplyThreshold' module and included the ‘Otsu Global’ and the ‘two classes’ options. Entropy was minimized and threshold correction factor for pixels was entered as ‘0.8’, and the lower and upper bounds on the threshold were ‘0.1’ and ‘1.0’, respectively. The 'MeasureImageAreaOccupied' module was used to measure surface areas of the defined CD68 and HE objects. DAB positive cells were identified using the 'IdentifyPrimaryObjects' module. The cells to be identified were defined within 8 and 40 pixels, objects outside this range were discarded. A three-class thresholding method was used with the method set to 'Otsu Global'. Entropy was minimized and the pixels in the middle intensity were set to the foreground. The threshold correction factor was set to 1 and the lower and upper bounds were set to '0.3' and '1' respectively. The method to distinguish clumped objects was set to 'Shape'.

*CellProfiler SMA pipeline*

This pipeline was used analyses the surface area and amount of SMA positive cells compared to HE surface area. Each image was processed using the ‘UnmixColors’ module allowing the DAB stain and hematoxylin stain to be extracted into two channels. DAB surface area and the HE surface area used similar 'IdentifyPrimaryObjects' module settings. Both included the ‘Otsu Global’ and the ‘three classes’ options. Entropy was minimized and pixels in the middle intensity class assigned to the foreground. The threshold correction factor was entered as ‘1.3’ and the lower and upper bounds on the threshold were ‘0.1’ and ‘1.0’ respectively. The method to distinquish clumped objects was set to 'none' and holes were filled in identified objects. The 'MeasureImageAreaOccupied' module was used to measure surface areas of identified SMA and HE objects.

We store CellProfiler measurements, like cell count, cell position, tissue surface area and other information in a database file (e.g. MySQL database or csv). Measurements can than be gathered and further analyzed using preferred statistical software, like R.


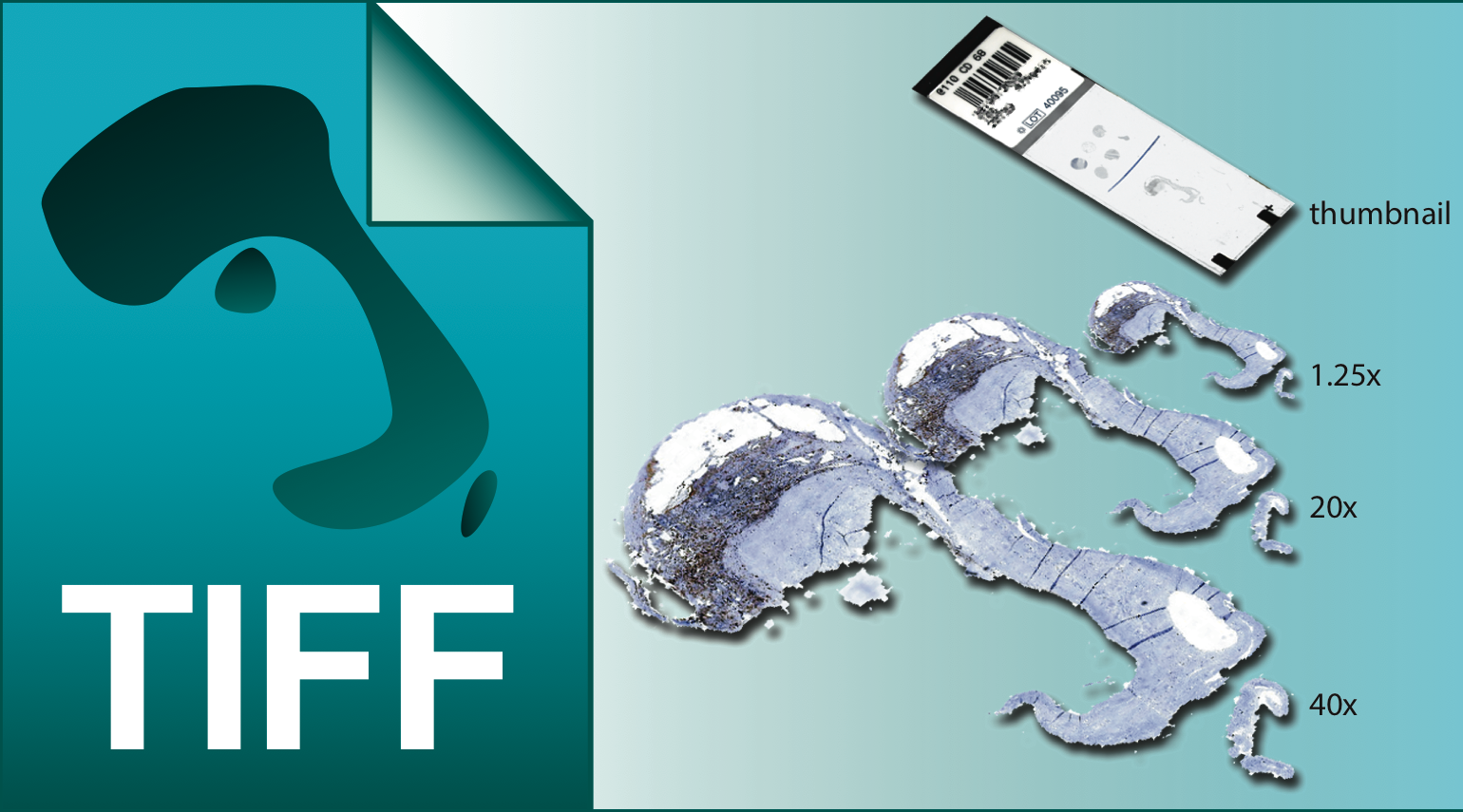


Figure 1. A visualisation of a multi-page pyramid TIFF file. This illustration shows a TIFF file with 4 layers (thumbnail, 1.25x, 20x, 40x), but virtual slides stored as TIFF files often contain up to 11 or more layers.

1. Vokes MS, Carpenter AE (2008) Using CellProfiler for automatic identification and measurement of biological objects in images. Curr Protoc Mol Biol Chapter 14: Unit14.17. doi:10.1002/0471142727.mb1417s82.
